# Hepatobiliary cancers in persons With HIV/AIDS in the United States

**DOI:** 10.1186/1750-9378-7-S1-O25

**Published:** 2012-04-19

**Authors:** Vikrant V Sahasrabuddhe, Meredith S Shiels, Katherine A McGlynn, Eric A Engels

**Affiliations:** 1 Division of Cancer Epidemiology and Genetics, National Cancer Institute, National Institutes of Health, Bethesda, MD, USA

## Background

Cancers of the hepatobiliary tract (liver, bile duct and gall bladder) are characterized by relatively infrequent occurrence, aggressive growth, and recurrences after treatment. Hepatocellular carcinoma (HCC) is of special concern in the context of HIV/AIDS due to substantial associated morbidity and mortality. The overall burden of liver cancer may increase in people with AIDS as the combined effects of alcohol use, coinfection with hepatitis C virus (HCV) and hepatitis B virus (HBV), and other risk factors manifest as chronic liver disease.

## Methods

Registry linkage data from the U.S. HIV/AIDS Cancer Match Study were used to estimate standardized incidence ratios (SIRs) to compare the risk of hepatobiliary cancers in people with HIV/AIDS to the general population. We also estimated rate ratios (RRs) of HCC by HIV risk group, calendar period and AIDS status. HIV risk groups were categorized by HCV prevalence [high prevalence: hemophiliacs, injection drug users (IDUs), and IDU-men who had sex with men (MSM); and low prevalence: heterosexuals, non-IDU MSM, and others].

## Results

**Figure 1:**
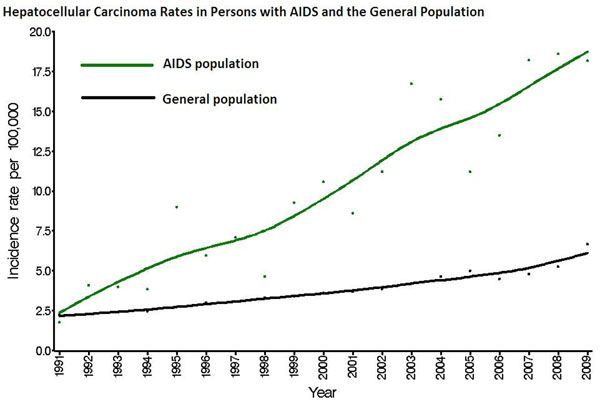


Compared to the general population, people with AIDS had higher risk for HCC (366 observed cases, SIR: 3.85; 95%CI: 3.47-4.27). SIRs were also elevated for other liver and intrahepatic bile duct tumors (27 cases, SIR: 3.26; 95%CI: 2.15-4.75), but not for cholangiocarcinomas (22 cases, SIR: 1.36; 95%CI: 0.85-2.05) or gallbladder tumors (11 cases, SIR: 1.39; 95%CI: 0.70-2.49). Adjusted for sex and age, people with high HCV prevalence were at higher risk for HCC than people with lower HCV prevalence (RR: 2.12; 95%CI: 1.72-2.60). SIRs were elevated for all HIV risk groups, with the highest SIRs among hemophiliacs (40.4), IDUs (5.59), and IDU-MSM (4.39). Steadily increasing risk among persons with AIDS was observed across calendar time, including during the HAART era (Figure 1)In an analysis limited to registry areas with data on HIV-infected people without AIDS), persons with AIDS had higher HCC risk than persons with HIV-only (RR: 2.59; 95% CI: 1.71-3.91).

## Conclusion

This study reinforces the primary role of HCV coinfection in HCC pathogenesis in persons with AIDS in the United States. HCC risk is higher in people with AIDS than people with HIV infection without AIDS, consistent with a contribution from prolonged immunosuppression. Rising HCC incidence in the era of HAART suggests that HAART itself does not fully correct the negative impact of HIV on HCV-related cirrhosis, and that access to appropriate anti-HCV therapies in HIV-infected individuals is critical for prevention of progression to HCC.

